# A Novel Digital Score for Abundance of Tumour Infiltrating Lymphocytes Predicts Disease Free Survival in Oral Squamous Cell Carcinoma

**DOI:** 10.1038/s41598-019-49710-z

**Published:** 2019-09-16

**Authors:** Muhammad Shaban, Syed Ali Khurram, Muhammad Moazam Fraz, Najah Alsubaie, Iqra Masood, Sajid Mushtaq, Mariam Hassan, Asif Loya, Nasir M. Rajpoot

**Affiliations:** 1 Department of Computer Science, University of Warwick, Coventry, CV47AL UK; 2 School of Clinical Dentistry, University of Sheffield, Sheffield, UK; 3 School of Electrical Engineering and Computer Science, National University of Sciences and Technology, H-12 Islamabad, Pakistan; 4 The Alan Turing Institute, NW1 2DB London, UK; 5 Department of Computer Science, Princess Nourah University, Riyadh, Saudi Arabia; 6 Shaukat Khanum Memorial Cancer Hospital Research Centre, Lahore, Pakistan; 7 University Hospitals Coventry, Department of Pathology, Warwickshire, UK

**Keywords:** Oral cancer detection, Oral cancer detection, Prognostic markers, Medical imaging, Disease-free survival

## Abstract

Oral squamous cell carcinoma (OSCC) is the most common type of head and neck (H&N) cancers with an increasing worldwide incidence and a worsening prognosis. The abundance of tumour infiltrating lymphocytes (TILs) has been shown to be a key prognostic indicator in a range of cancers with emerging evidence of its role in OSCC progression and treatment response. However, the current methods of TIL analysis are subjective and open to variability in interpretation. An automated method for quantification of TIL abundance has the potential to facilitate better stratification and prognostication of oral cancer patients. We propose a novel method for objective quantification of TIL abundance in OSCC histology images. The proposed TIL abundance (TILAb) score is calculated by first segmenting the whole slide images (WSIs) into underlying tissue types (tumour, lymphocytes, etc.) and then quantifying the co-localization of lymphocytes and tumour areas in a novel fashion. We investigate the prognostic significance of TILAb score on digitized WSIs of Hematoxylin and Eosin (H&E) stained slides of OSCC patients. Our deep learning based tissue segmentation achieves high accuracy of 96.31%, which paves the way for reliable downstream analysis. We show that the TILAb score is a strong prognostic indicator (*p* = 0.0006) of disease free survival (DFS) on our OSCC test cohort. The automated TILAb score has a significantly higher prognostic value than the manual TIL score (*p* = 0.0024). In summary, the proposed TILAb score is a digital biomarker which is based on more accurate classification of tumour and lymphocytic regions, is motivated by the biological definition of TILs as tumour infiltrating lymphocytes, with the added advantages of objective and reproducible quantification.

## Introduction

In 2014, there were more than 11,000 instances of Head and Neck (H&N) cancers in the UK and more than 2,300 deaths resulting from oral cavity cancers^1^. Oral squamous cell carcinoma (OSCC) is the most common malignancy of the H&N region^2^ in both males (42%) and females (46%). The OSCC prevalence is almost twice as common in males and 3 times more in females than the next most common cancer which is larynx SCC (26% in males, 13% in females)^3^. OSCC is associated with invasion and destruction of local tissues and maxillofacial bones with significant associated morbidity. In addition to early recurrence, frequent lymph nodes metastasis and extranodal extension^4^ are further challenges in the management of OSCC patients. The high morbidity and mortality rates in OSCC patients^5,6^ highlight the need for an objective and quantitative analysis of any potential prognostic markers to help identify tumours which may respond poorly to therapy^7^.

**Figure 1:**
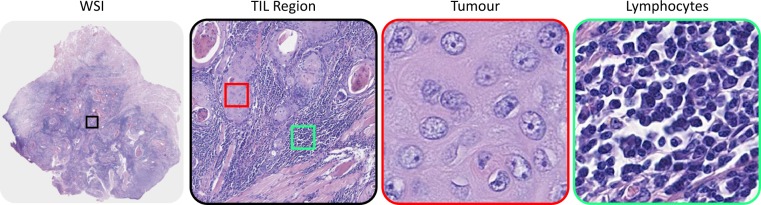
An example image of TIL region (black) in a Whole Slide Image. High resolution view of tumour (red) and lymphocyte (green) regions are shown in separate images.

Tumour -infiltrating lymphocytes (TILs) have been analysed in a wide range of cancers with strong evidence demonstrating their prognostic value as a supplement to the Tumour -Node-Metastasis (TNM) staging^8–10^. TILs mainly comprise T lymphocytes which migrate from the blood into the tumour as part of the body’s immune ‘fight-back- response’. However, it is important to analyse these cells in the correct context. A large number of lymphocytes can be present in inflamed and cancerous tissues and, therefore, it is vital to develop methods to specifically analyse lymphocytes infiltrating the tumour as these are the ones that are likely to be of prognostic significance^11–13^. These areas can be referred to as the TIL regions where both tumour and lymphocytes are co-localized (as illustrated in Fig. 1). Numerous studies have reported the correlation of TIL density with improved overall survival (OS) and longer disease-free survival^14,15^. It has been shown that the quantification of spatial patterns of TILs in the tumour regions can have prognostic value significantly supplementing or even superseding the TNM staging in certain settings^16,17^. However, the currently used method of visual TIL quantification is subjective with inter- and intra-observer variability and lack of diagnostic reproducibility^18^. Therefore, it is imperative to develop an automated method for objective quantification of TILs in OSCC to address these challenges.

Digitization of histology slides has recently ignited interest from computer scientists to develop techniques for the automated analysis of digitized multi-gigapixel whole slide images (WSIs) of tissue slides^19–23^. The approval of digitized WSIs as a primary histopathological diagnosis tool by the US Food and Drug Administration (FDA)^24^ has further boosted this interest. The digitization of WSIs offers unique opportunities to quantify vast amounts of information about morphological and spatial architectural patterns in WSIs and to discover novel prognostic and predictive histological markers to potentially improve cancer treatment^25^. For instance, it has recently been shown by Saltz *et al*.^16^ that a digital marker based on TIL spatial patterns carries prognostic significance for multiple cancer tissue types.

**Figure 2:**
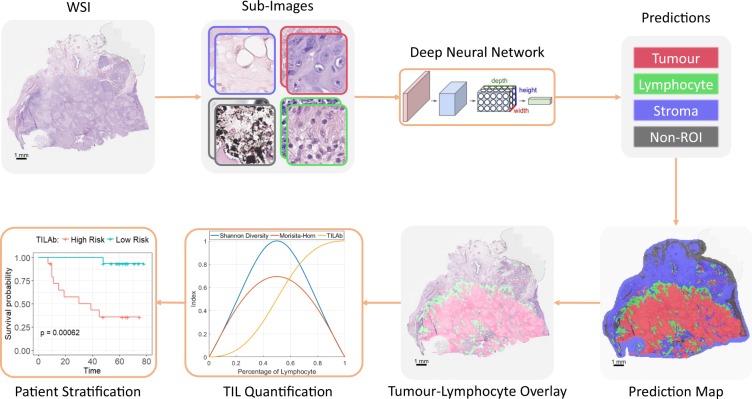
Flow diagram of the proposed method. Colour represents the respective class.

In this paper, we present a novel method to obtain an automated TIL abundance score and explore its prognostic significance for Disease Free Survival (DFS) of OSCC patients. The proposed approach, as illustrated in Fig. 2, comprises of three main components: WSI segmentation into biologically significant tissue phenotypes, identification and quantification of TILs, and their prognostic analysis. First, WSI segmentation into biologically significant tissue phenotypes is modelled as an image patch based tissue classification problem. Different tissue regions such as tumour, lymphocytes, and stroma in the WSI are classified using a deep convolutional neural network (CNN). Second, a tumour -lymphocyte co-localization based binary classifier is developed using statistical co-localization measures for detecting the presence or absence of TILs in OSCC tissue slides. The extent of lymphocytic infiltration, which we term as the TIL Abundance (TILAb) score, in tumour region is quantified by a combination of lymphocyte-to-tumour ratio and their statistical co-localization. Finally, the prognostic significance of the TILAb score for DFS is investigated by employing univariate and multivariate analysis. To the best of our knowledge, there is no existing method for automated quantification of TIL Abundance from digitized WSIs and the downstream analysis of OSCC patient survival. We show that the TILAb score is a strong prognostic indicator of DFS in OSCC patients in agreement with previous findings based on manual TIL quantification^26^. Our main contributions in this paper are as follows:

- A methodology for segmentation of biologically significant regions in OSCC tissue is presented which includes segmentation of tumour areas, lymphocytes, stromal regions, and artefacts in a WSI.
- We propose a novel scoring of TIL abundance, termed as the TILAb score, to quantify the extent of lymphocytic infiltration in the tumour region which is a combination of lymphocyte-to-tumour ratio and their statistical co-localization in a WSI.
- The reproducibility and objectivity of the TILAb score are investigated in two different ways: First, by analyzing the consistency between statistical co-localization based TIL detection and a pathologist’s detection. Second, by evaluating the prognostic significance of TILAb score for DFS of OSCC patients.

## Materials and Methods

Ethical approval was obtained from the institutional review board (Ref. No. 17-02-17-10) at Shaukat Khanum Memorial Cancer Hospital and Research Centre (SKMCH&RC) and National Bioethics Committee (No.4-87/17/NBC-234-Exempt/NBC/2592), Pakistan. All methods and experiments were carried out in accordance with relevant guidelines and regulations. Exemption from written consent was granted by the IRB at SKMCH&RC and NBC on grounds that the data and images used in the study were already in existence and were collected and reported in an anonymized way ensuring confidentiality of participants. The study did not involve any intervention or interaction with the participants. The research involved no more than minimal risk to the participants and involves no procedures for which written consent is normally required outside of the research context and the waiver did not adversely affect the rights and welfare of the participants.

### Patient selection

Patients with OSCC diagnosed between 2010–11 at SKMCH&RC were selected from the electronic medical records (hospital information system). SKMCH&RC follows a multidisciplinary approach, therefore all patients were treated by both a radiation oncologist and by a H&N surgeon. Cases included both primary and recurrent tumours that underwent complete tumour resection with or without lymph node dissection and for which at least three year survival data were available. After the initial review of data, 60 patients were selected out of 155 oral cancer cases as per the study protocol (see Fig. S2 in the Supplementary Materials). The cases excluded were those with either an incomplete resection and those where survival follow up was less than 3 years. A final cohort of 60 malignant cases and 10 controls were finalized where the control cases did not suffer from OSCC. Formalin fixed paraffin embedded blocks were retrieved and representative slides from each case were reviewed by the study pathologists (AL and SM) and confirmed to be OSCC. In addition, slides were also reviewed for additional histopathological features such as patterns of invasion, TILs and perineural invasion. For this study, oral cavity cancers were defined as carcinomas of the mouth including lip, tongue, cheeks, floor of the mouth, hard and soft palate, whereas tumours of the salivary glands were excluded. After compilation of the clinical and pathologic information, including American Joint Committee on Cancer (AJCC) 7th edition stage, clinical and pathological information was retrieved from the electronic medical records, as summarized in Table 1. De-identified, tissue slides were digitally scanned in University Hospitals Coventry & Warwickshire using Omnyx Integrated Digital Pathology system at 40× magnification with a resolution of 0.275 *μm* per pixel. There were 193 tissue sections in 70 digitally scanned WSIs as many WSIs contain multiple tissue sections.

**Table 1:** Summary of clinical parameters of the OSCC Cohort.

Clinical Parameters		Full Cohort	Modelling Set	Test Set
No. of Patients		60	30	30
Age (years)		49.77 ± 10.99	50.57 ± 9.77	48.97 ± 12.03
Survival (months)	Overall	54.78 ± 17.91	56.00 ± 17.75	53.47 ± 17.96
	Disease Free	48.87 ± 22.51	50.00 ± 22.44	48.00 ± 22.55
Gender	Male	36	17	19
	Female	24	13	11
Node-Stage	I/II	25	17	8
	III/IVa	35	13	22
Grade	I/II	48	23	25
	III	12	7	5
Growth Patterns	Type 1/2	21	13	8
	Type 3/4	39	17	22
TILs	Absent/Mild	32	17	15
	Moderate/Severe	28	13	15
Patient Status	Alive	47	24	23
	Dead	13	6	7
Disease Recurrence	Yes	19	9	10
	No	41	21	20

### Patient characteristics

Our study cohort consists of 70 cases including 60 OSCC and 10 control cases. DFS information was available for all the malignant cases where survival time was calculated from the date of surgery. DFS was censored at the date of first recurrence or death, whichever occurred first, or the date of last contact for the patients alive and without recurrent disease. The follow up period ranged from 3.8 years to a maximum of 6.10 years at the time of data retrieval (2017). Median DFS was 58 months (range, 4–86 months) and median age 50 years (range, 25–75 years). Approximately 32% (*n* = 19) of patients suffered from disease recurrence whereas 22% (*n* = 13) had died by the time of data retrieval. About 60% (*n* = 36) of the patients were male, while 42% (*n* = 25) are at stage I/II and remaining are at stage III/IVa. Further details of all clinical parameters are given in Table 1.

### Pathologist annotations

We split our OSCC cohort into two equal sized subsets, one for modelling and the other for test. Six cases from the modelling set were considered for validation of the proposed tissue region classifier. An oral and maxillofacial pathologist (SAK) reviewed all the digitized WSIs and marked the ground truth at two different level of abstraction: TIL presence or absence on all the slides, and tumour /lymphocytic regions on the modelling subset. At high level, the presence or absence of TILs in 193 tissue section from all WSIs was marked where 111 were TIL positive (*T*^+^) and 82 TIL negative (*T*^−^). For the classification of biologically significant regions, more than half million regions (belonging to different classes such as tumour, lymphocytes, and stroma) were marked in all WSIs of the modelling cohort. The annotations were then used for training and validation of the proposed method.

### Whole slide image analysis

Whole slide images are multi-gigapixel images and cannot be used directly for image analysis tasks particularly training a deep learning based classifier. Therefore, we divide the WSIs into small regions (patches) for processing. A deep learning based classifier (see next section) is applied on the patches to identify whether the patch contains tumour, lymphocytes or other histological primitives. However, the regions where the lymphocytes are infiltrating the tumour may not be confined within a patch. Besides, there is considerable variation in the size of TIL regions, making the quantification of TILs a non-trivial task. We address this issue by adopting the widely accepted definition of TILs, i.e., lymphocytes that lie in the neighbourhood of tumour areas. The patch labels predicted as lymphocytes or tumour are then used to compute a statistical measure of co-localization, which is further incorporated into the computation of the proposed score of lymphocytic infiltration, i.e. the TILAb score.

### Tissue region classifier

**Figure 3:**
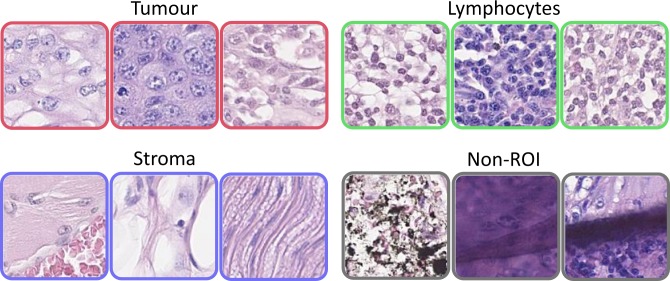
Exemplar patches of tumour, lymphocyte, stroma, and Non-ROI (artefacts) classes.

A tissue section in a WSI contains many different types of cells and regions, such as tumour cells, lymphocytes, and stromal regions (i.e. fibroblasts, endothelial cells, blood vessels, muscle, fat and red-blood cells). A WSI may also contain slide preparation and scanning artefacts, such as tissue folding and blurring, which need to be ignored. Therefore, we classify OSCC tissue sections into biologically significant regions. Tumour and lymphocyte rich regions are important for the detection and quantification of TILs. Precise classification of stromal regions along with other lymphocytic regions is necessary to discriminate between TILs and regular lymphocytes that do not lie within the vicinity of tumour regions. The fourth and final class of regions consists of scanning and tissue artefacts, which are labelled as non-regions of interest (Non-ROIs). We select a reasonably small patch size (128 × 128 pixels at 20×) such that we do not require precise pixel-level tissue segmentation. Figure 3 shows three samples of each of the 4 class labels: tumour, lymphocytes, stroma, and Non-ROIs.

Deep learning models have significantly improved the state-of-the-art in many natural image based problems such as visual object detection and recognition^27,28^ and scene labeling^29^. Most popular deep learning networks for the classification task are ResNet^30^, DenseNet^31^, Inception^32^, Xception^33^ and MobileNet^34^. Each network shows competitive results on one of the largest image classification datasets, ImageNet^28^. We train these networks for tissue classification task to get a strong baseline model. We extracted 400,000 patches for training and 100,000 patches for validation from WSIs. Both training and validation datasets have equal numbers of patches for each class. During training, we leverage online data augmentation with random rotation of 0, 90, 180 or 270 and random flipping. We select the best model of each classifier after training it for at least 125,000 optimization steps with RMSProp optimizer. During testing, the patch classifier takes non-overlapping patches from a WSI and outputs probabilities of all classes for each patch, resulting in a probability map at the WSI level. The probability maps are converted into prediction maps by selecting the class with the highest probability for each patch (Fig. 2). The prediction map at the WSI level is eventually used for TIL identification and for computation of the TILAb score.

### TIL quantification

**Figure 4:**
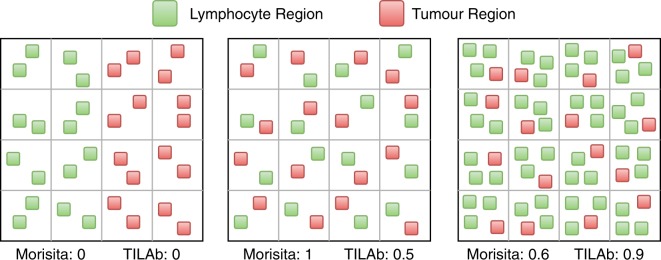
The illustration of tumour and lymphocyte co-localization patterns, each image is divided into 4 × 4 grids. (Left) Highly segregated appearance of tumour and lymphocytic regions. (Center) Fully co-localized regions. (Right) Lymphocyte rich co-localization.

Objective quantification of TILs is a non-trivial task as it depends on the co-localization of both tumour and lymphocytic regions. In ecology, co-localization of different species is used to understand their community structure^35,36^. Tumour and lymphocytes in TIL regions could be considered as two different interacting species in the histological landscape. We consider WSI areas containing tumour and/or lymphocytes and investigate the utility of co-localization score of tumour and lymphocytes in the WSI. For this purpose, a WSI is divided into *m* × *n* equal sized grids, such that the grid size is greater than the size of input patch for the region classifier. The co-localization score *M* in terms of the Morisita-Horn^37^ index is then defined as follows,

1 $$M=\frac{2{\sum }_{i=1}^{m}{\sum }_{j=1}^{n}({p}_{ij}^{l}\times {p}_{ij}^{t})}{{\sum }_{i=1}^{m}{\sum }_{j=1}^{n}{({p}_{ij}^{l})}^{2}+{\sum }_{i=1}^{m}{\sum }_{j=1}^{n}{({p}_{ij}^{t})}^{2}},$$

where ${p}_{ij}^{l}$ and ${p}_{ij}^{t}$ represent the percentage of lymphocytic and tumour regions in the (*i*, *j*)^*th*^ grid-cell, respectively (Fig. 4). If a grid-cell does not contain any tumour and lymphocytic region, then it would not contribute towards the co-localization score. However, if a grid-cell only contains one type of region, either tumour or lymphocyte, then it only contributes to the denominator of the equation thus results in a relatively small value of the co-localization score. If all the grid-cells contain only unique type of regions, then the co-localization score become zero. This score ranges from 0 to 1 and the score is maximum when each of the grid-cells has exactly the same number of tumour and lymphocyte patches, as shown in Fig. 4.

**Figure 5:**
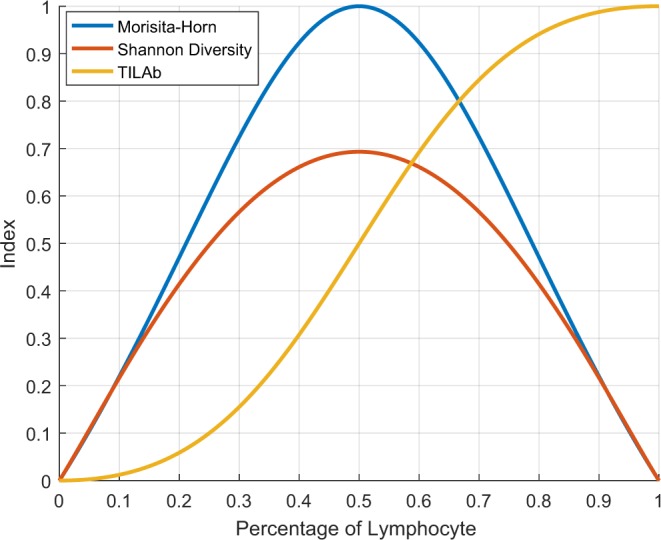
Plots of Morisita-Horn, Shannon diversity and TILAb indices for different percentages of lymphocyte. At each point, the percentage of tumour is equal to 1 – percentage of lymphocyte.

We also consider the Shannon diversity index^38^ to quantify the co-localization of tumour and lymphocytic regions in a WSI. It computes the diversity of two classes in a given region that is also aligned with the above definition of TILs. For *m* × *n* grids of equal sizes in a WSI, the Shannon diversity index, *S*, is defined as follow,

2 $$S=\frac{-{\sum }_{i=1}^{m}{\sum }_{j=1}^{n}({p}_{ij}^{l}\times \,\mathrm{ln}\,{p}_{ij}^{l}+{p}_{ij}^{t}\times \,\mathrm{ln}\,{p}_{ij}^{t})}{m\times n},$$

where ${p}_{ij}^{l}$ and ${p}_{ij}^{t}$ represent the percentage of lymphocytic and tumour regions in the (*i*, *j*)^*th*^ grid-cell. The co-localization computed using Shannon diversity index is relatively smaller in magnitude as compared to the Morisita-Horn index with the maximum value of 0.7 at the maximum co-localization point of tumour and lymphocytic regions (Fig. 5).

### The proposed TILAb score

The Morisita-Horn and Shannon diversity are two objective and efficient measures for quantification of co-localization. However, these methods give equal importance to all the constituent classes (or species), which consequently results in a symmetric co-localization score (as can be seen in Fig. 5). For instance, 20% lymphocytes and 80% tumour in a region will give the same co-localization score as another region consisting of 80% lymphocytes and 20% tumour. However, the lymphocyte proliferation in tumour is considered to be good prognostic indicator for patient survival. Therefore, the symmetric nature of these measures is not ideal for obtaining an objective TIL abundance score of prognostic importance. We proposed the TIL abundance (TILAb) score, *T*, which is a combination of the lymphocyte-to-tumour ratio and their co-localization, as defined below,

3 $$T=(\begin{array}{cc}\frac{C}{2}\times \frac{{\sum }_{i=1}^{m}{\sum }_{j=1}^{n}({p}_{ij}^{l})}{{\sum }_{i=1}^{m}{\sum }_{j=1}^{n}({p}_{ij}^{t})}, & {\rm{i}}{\rm{f}}\,{\sum }_{i=1}^{m}{\sum }_{j=1}^{n}({p}_{ij}^{t}) > 0\\ 1, & {\rm{o}}{\rm{t}}{\rm{h}}{\rm{e}}{\rm{r}}{\rm{w}}{\rm{i}}{\rm{s}}{\rm{e}}\end{array})$$

where C is a co-localization measure and M or S can be used as a co-localization measure. Right half of the above equation shows the lymphocytes-to-tumour ratio in WSI. We normalize the range of TILAb score between 0 to 1 by dividing it by a factor of 2. The proposed TILAb score objectively quantifies the TILs and its formulation is generic enough to work with both Morisita-Horn and Shannon diversity indices. Figure 5 shows the distribution of TILAb score using Morisita-Horn index based co-localization at different percentages of lymphocytes. It can be seen that both Morisita-Horn and Shannon diversity indices have relatively small values even with high lymphocytic percentage, whereas the TILAb score increases with the increase in lymphocytic infiltration (See Supplementary Section 1). Moreover, TILAb score remains same for different tumour and lymphocyte density with same ratio (See Fig. S3).

### Survival analysis

The TILAb score based statistical analysis is performed for DFS in order to demonstrate its prognostic significance as an independent biomarker. Kaplan-Meier^39^ and Cox proportional-hazards model^40^ are used for survival and hazard analyses, respectively. To stratify patients into high-risk (short-term survival) and low-risk (long-term survival) groups, we find an optimal cut-point on the TILAb score value from the modelling subset where the statistical significance of the difference in DFS between the two groups is the largest. Log-rank test based *p* value is used to assess the statistical significance of the survival stratification observed between cases predicted to be short-term and long-term DFS where *p* < 0.05 is considered significant. For multivariate analysis, Cox proportional-hazards model is used which simultaneously evaluates the effect of several factors on survival. We report the hazard ratio along with lower and upper 95% confidence interval. Global statistical significance of the model is measured by the Wald test.

### Implementations

We made use of the open source Keras and Tensorflow libraries to train our tissue region classifier network. All the training is done on Scan 3XS computer with Intel(R) Xeon(R) processor (E5-2690) and two NVIDIA TitanX 12GB GPUs. The trained model is then used to get the probability maps of WSI images. The lymphocytes and tumour probability maps are used to compute the TILAb score according to the Eq. (3). All the statistical analysis including p-values, c-index, Kaplan-Meier and Cox proportional-hazards analysis are done using standard R packages i.e. *survival, cvAUC, ROCR, and survMisc*.

## Results

### Tissue region classification

In order to get a strong baseline multi-class Tissue Region Classifier (TRC) model, we employ five state-of-the-art convolutional neural network models (ResNet50^30^, DenseNet^31^, Inception^32^, Xception^33^ and MobileNet^34^), denoted as TRC-1 to TRC-5 respectively. Table 2 gives the quantitative performance of these TRCs for multi-class patch level classification on the validation dataset; details of the comparative performance analysis can be found in Table S1 in the Supplementary Materials. Among the five classifiers, MobileNet (TRC-5) shows superior performance as compared to the other networks. It is a light-weight network that leverages separable convolutions to reduce the number of required parameters and computations, resulting in consumption of relatively less memory and computational resources and making it an attractive choice for the processing of multi-gigapixel whole slide images. For evaluation of proposed approach, we used both the best performing (TRC-5) and the least performing (TRC-1) tissue region classifiers for downstream analysis.

**Table 2:** Quantitative performance of five different tissue region classifiers on validation dataset of 10^5^ patches.

Classifiers	Accuracy	Sensitivity	Specificity	F1-Score	AUC
TRC-1 (ResNet50^30^)	94.04	88.19	96.03	88.08	97.88
TRC-2 (DenseNet^31^)	95.40	90.80	96.94	90.78	98.56
TRC-3 (Inception-v3^32^)	95.84	91.91	97.25	91.70	98.86
TRC-4 (Xception^33^)	95.94	92.16	97.32	91.88	98.83
TRC-5 (MobileNet^34^)	**96.31**	**92.66**	**97.55**	**92.62**	**98.91**

**Figure 6:**
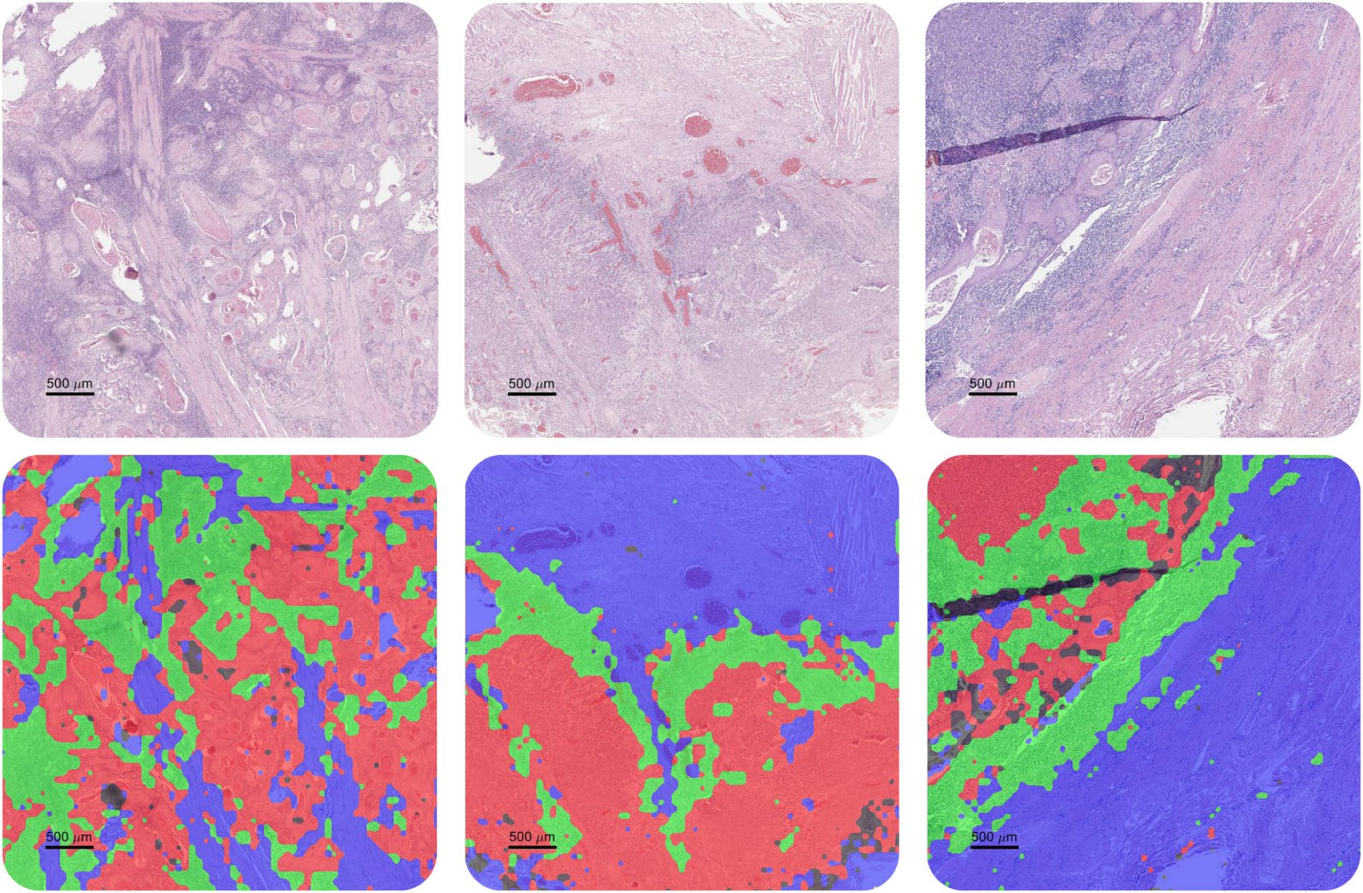
Tissue region classification results by TRC-5 where tumour, lymphocytic, stromal and non-ROI regions are represented by red, green, blue and black colours, respectively.

Sample visual results for tissue region classification obtained with TRC-5 are shown for illustration in Figs 6 and S4, where tumour, lymphocytic, stromal and non-ROI regions are shown in different colours â€“ see detailed confusion matrix in Fig. S5 in the Supplementary Materials. Lymphocytic regions are classified with the highest accuracy whereas tumour and non-ROI regions show slight overlap. In general, TRC-5 gives the best classification performance, as can also be seen in the Precision-Recall curve in Fig. S6, which shows relatively high values of True Positive Rate (TPR) and low values for False Positive Rate (FPR) for both tumour and lymphocyte areas.

### TIL quantification and TILAb score

**Figure 7:**
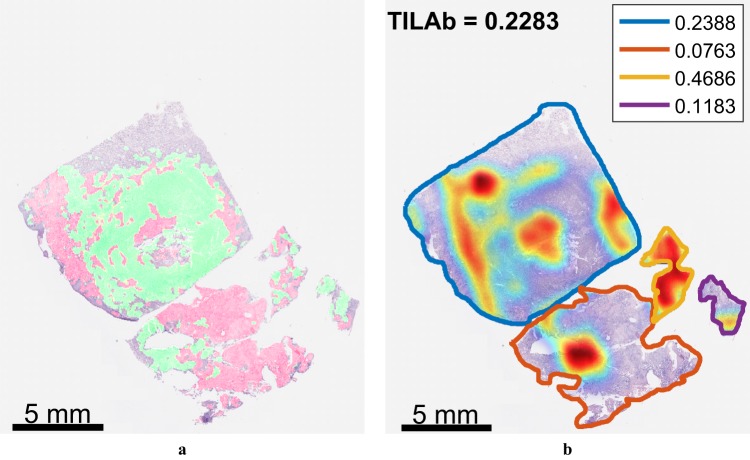
(**a**) Whole slide image at low resolution (1.5×) with tumour and lymphocytic region predictions overlaid in red and green colours, respectively. (**b**) Tumour -lymphocyte co-localization map along with co-localization score for each tissue section in upper right corner and WSI level TILAb score. Colour codes map the co-localization score to respective tissue sections.

The score for co-localization of tumour and lymphocytes for each tissue section is computed using the Eq. (1). The TILAb score at WSI level is also computed according to Eq. (3) and later used for the survival analysis. Figures 7 and S7 show the co-localization scores of different tissue segments in the WSI along with the WSI level TILAb score. For evaluating the TILAb score’s performance with manual marking, tissue segments on a WSI are marked for the presence (*T*^+^) and absence (*T*^−^) of TILs by an expert pathologist (SAK). Five different performance measures are used to evaluate the performance of the proposed method, as shown in Table 3. Co-localization score based on the best performing region classifier (TRC-5) achieved 88.96% Area Under the Curve (AUC). It is pertinent to mention that the least performing region classifier (TRC-1) also performed reasonably well with 87.54% AUC.

**Table 3:** Performance of tissue section classification into TIL positives and negatives.

Classifiers	Accuracy	Sensitivity	Specificity	F1-Score	AUC (95% CI)
TRC-1	80.19	85.45	74.51	81.74	87.54 (80.95–94.12)
TRC-5	79.05	79.69	78.05	82.26	88.96 (82.68–95.13)

### Survival analysis

**Figure 8:**
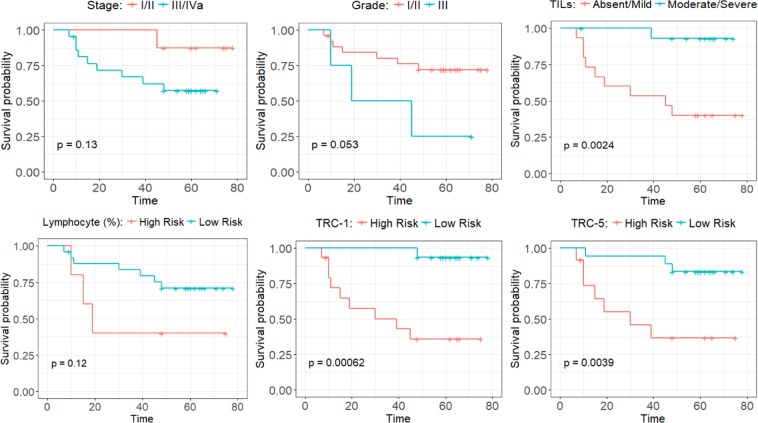
Kaplan Meier (KP) curves for disease free survival of OSCC on test subset. Top row contains the KP curves for pathological parameters (stage, grade, and manual TIL quantification) whereas bottom row shows the KP curves of digital parameters (Lymphocyte percentage in WSI, TILAb score using TRC-1 and TRC-5). The optimal cut-point values for digital parameters are 0.017, 0.124 and 0.137, respectively.

The prognostic significance of TILAb score for DFS is investigated using Kaplan-Meier (KM) curves and Cox hazard analyses by conducting univariate and multivariate analysis of digital, clinical, and pathological parameters. We propose the TILAb score based prognostic model to predict the binary outcome of DFS. The prognostic model finds the optimal cut-off point for TILAb score on the modelling subset and uses that cut-off on the test subset for binary prediction. The proposed TIL quantification method contains one hyper-parameter which is the size of a grid-cell. We experimented with eight different neighbourhood sizes on tissue section to investigate their impact on survival analysis. The size of the smallest grid-cell is 0.28 *mm* × 0.28 *mm* and we used a fixed step size of 0.14 *mm* for increment in grid-cell size, up to the largest grid-cell of size 1.2 *mm* × 1.2 *mm*. Table 4 shows that our proposed TILAb score is statistically significant for all sizes with both best and least performing tissue region classifiers (TRC-1 and TRC-5). However, the standard non-normalized Morisita-Horn and Shannon diversity measures show significant results only for the smallest grid-cell size when using the best performing region classifier (TRC-5). This indicates the significance of TILAb score for DFS analysis. Moreover, the high concordance indices (c-indices) of the prognostic models are also evidence of the predictive ability of proposed models. Tables 5 and S2 (in Supplementary Materials) lists the c-index value for both DFS and OS models. Models for DFS achieve high c-index value as compare to the models for OS. On the other hand, models with different colocalization methods (TILAb-MH, TILAb-SD) show similar results within DFS and OS. Kaplan-Meier curves in Fig. 8 show that the proposed TILAb score is significantly associated with long term (low risk) DFS of OSCC patients (*p* = 0.00062). However, the lymphocytic percentage in a WSI without any correlation with tumour does not show any statistical significance. The proposed digital TILAb score has higher statistical significance as compared to the manual TIL score given by expert pathologists (SM and AL) after visual inspection. Kaplan-Meier curves for other clinical and pathological parameters are shown in Fig. S8.

**Table 4:** Comparison of the different TIL quantification methods based on their prognostic significance (logrank test based *p*-values) at different grid-cell sizes (smallest to largest).

Region Classifier	Quantification Methods	1	2	3	4	5	6	7	8
TRC-1	Morisita-Horn (MH)	0.1590	0.1610	0.1560	0.3250	0.2340	0.4760	0.4760	0.4760
	Shannon Diversity (SD)	0.1590	0.1610	0.1610	0.3250	0.2190	0.6550	0.4760	0.4760
	TILAb-MH	**0.0146**	**0.0258**	**0.0146**	0.0012*	**0.0258**	0.0006*	0.0020*	0.0006**
	TILAb-SD	**0.0146**	**0.0146**	**0.0258**	**0.0146**	**0.0258**	**0.0258**	0.0006**	0.0006**
TRC-5	Morisita-Horn (MH)	**0.0416**	0.0666	0.1030	0.1800	0.0666	0.1800	0.2340	0.1790
	Shannon Diversity (SD)	**0.0416**	0.0666	0.0666	0.1160	0.0666	0.1800	0.2340	0.1790
	TILAb-MH	**0.0191**	**0.0191**	0.0077*	0.0077*	**0.0258**	**0.0236**	0.0020*	0.0038*
	TILAb-SD	**0.0191**	**0.0359**	**0.0146**	**0.0258**	**0.0110**	**0.0146**	0.0020*	0.0020*

[a] Significance codes: **0.05**, 0.01*, 0.001**.

**Table 5:** C-Indices (with 95% CI) of TRC-1 and TRC-5 based prognostic models for DFS at different grid-cell sizes (smallest to largest).

Grid-Cell	TRC-1						TRC-5					
	TILAb-MH			TILAb-SD			TILAb-MH			TILAb-SD		
	C-Index	Lower CI	Upper CI	C-Index	Lower CI	Upper CI	C-Index	Lower CI	Upper CI	C-Index	Lower CI	Upper CI
1	0.8700	0.7489	0.9911	0.8700	0.7489	0.9911	0.8650	0.7297	1.0000	0.8650	0.7297	1.0000
2	0.8700	0.7497	0.9903	0.8650	0.7416	0.9884	0.8450	0.7093	0.9807	0.8450	0.7079	0.9821
3	0.8550	0.7265	0.9835	0.8500	0.7182	0.9818	0.8400	0.7020	0.9780	0.8500	0.7167	0.9833
4	0.8400	0.6999	0.9801	0.8500	0.7182	0.9818	0.8450	0.7086	0.9814	0.8450	0.7086	0.9814
5	0.8450	0.7086	0.9814	0.8450	0.7065	0.9835	0.8350	0.6921	0.9779	0.8400	0.6999	0.9801
6	0.8300	0.6825	0.9775	0.8350	0.6894	0.9806	0.8400	0.6999	0.9801	0.8350	0.6908	0.9792
7	0.8300	0.6825	0.9775	0.8300	0.6825	0.9775	0.8350	0.6934	0.9766	0.8250	0.6750	0.9750
8	0.8200	0.6696	0.9704	0.8250	0.6725	0.9775	0.8400	0.6992	0.9808	0.8500	0.7160	0.9840

**Figure 9:**
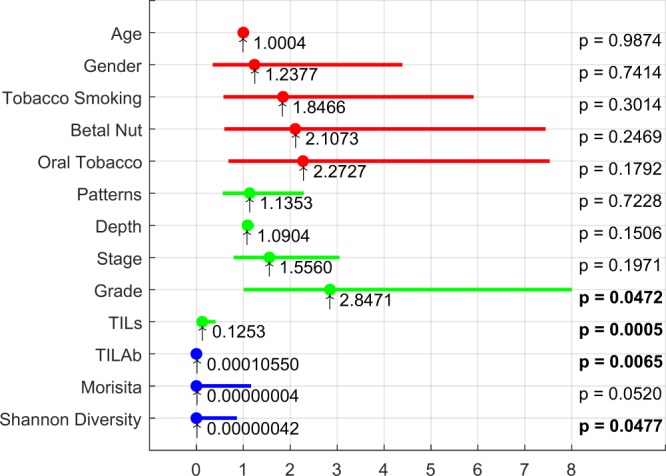
Univariate analysis for clinical (red), pathological (green) and digital (blue) parameters. Hazard ratios are represented by a filled circle whereas edges of each line represent the lower and upper confidence interval of 0.95%. p-value using Wald test is shown on the right end for each parameter. Digital parameters are computed using TRC-5 predictions.

Results of the univariate analysis of prognostic significance of digital, clinical and pathological parameters on the test subset are shown in Fig. 9. We employed Cox proportional hazards method for univariate analysis for both quantitative and categorical predictor variables. The clinical parameters do not show any significant correlation with DFS, with the confidence interval range of hazard ratios (lower and upper 95% bounds) being quite large, except for age. The pathological parameters show better association with DFS as compared to clinical parameters especially tumour grade and manual quantification of TILs. Among digital scores, the proposed TILAb score is shown to be statistically significant (*p* = 0.0065) with hazard ratio of 0.0001 (1.446 × 10^7^ − 0.0769). We also investigate the prognostic value of the TILAb score in the context of other pathological parameters such as grade, stage and patterns, as shown in Table 6. For this purpose, we conduct the multivariate Cox proportional hazards analysis using the TILAb score adjusted by other histological features, as shown in Table 6. TILAb score is significant when combined with pathological features. Additionally, TILAb score is also significant when combined with grade, stage or pattern.

**Table 6:** Multivariate analysis of TILAb score along with other clinical parameters. TILAb score is computed with the Morisita-Horn as co-localization measure on TRC-5 predictions, while the *p*-value is computed using the Wald test.

	*p*	HR	Lower 95%	Upper 95%
	A - Overall Significance (**0.0334**)			
TILAb	**0.0103**	3.423e05	1.321e08	0.0887
Grade	0.3625	1.9720	0.4574	8.5003
Stage	0.2307	1.5280	0.7637	3.0587
Pattern	0.9387	1.0370	0.4105	2.6196
	B - Overall Significance (**0.0090**)			
TILAb	**0.0085**	6.701e5	5.232e08	0.0858
Grade	0.0745	2.3810	0.9177	6.1798
	C - Overall Significance (**0.0128**)			
TILAb	**0.0061**	3.267e05	2.044e08	0.0522
Stage	0.1377	1.6610	0.8499	3.2466
	D - Overall Significance (**0.0105**)			
TILAb	**0.0038**	2.243e05	1.610e08	0.0313
Pattern	0.1324	1.6750	0.8555	3.2803

## Discussion and Conclusion

The presence of lymphocytes in the vicinity of tumour cells has been reported to carry high prognostic value^14,15^. Quantification of TILs can not only significantly supplement the clinical cancer staging information but could be used as an accurate predictor of disease progression^8–10^. The abundance of TILs in a tissue slide (or its digitized WSI) indicates the host immune response against cancer and/or response to treatment. The density and spatial arrangement of TILs is correlated with improved overall survival (OS) and longer DFS. However, the manual quantification of TIL abundance is subjective, leading to inter-/intra- observer variability and lacking diagnostic reproducibility.

We propose a deep learning based approach for identification and quantification of TILs in OSCC cases. A digital score of TIL Abundance is computed and its prognostic potential is investigated for DFS in OSCC patients. The biologically significant regions in tissue such as tumour, lymphocytes, and stroma are classified using convolutional neural network. Several techniques are available in literature for detection and classification of histological structures in WSI images^19,41–45^. However, very few are used for downstream prognostic analysis for DFS. In this study, the results of tumour and lymphocytic region classification are used to compute the TIL abundance score (TILAb) followed by its evaluation as a prognostic marker. For tissue region classification, we experiment with different state-of-the-art CNN based image classifiers. We have chosen the classifiers giving the highest (TRC-5) and the lowest (TRC-1) patch level classification accuracy for further analysis of TIL detection, computing the abundance score and survival analysis. The results obtained by both of the classifiers are statistically significant.

Detection of clinically significant histopathological regions is followed by the detection and quantification of TILs. In this regard, Saltz *et al*.^16^ have studied the correlation between TIL spatial organization and molecular characteristics in histology images. They have used CNN for detection of lymphocytes in tissue regions, which they referred to as TILs. In contrast, we considered only those lymphocytes as TILs that have infiltrated into (or are co-localized with) tumour regions. Moreover, Saltz *et al*. used affinity propagation algorithm to identify/quantify the local spatial patterns in the detected lymphocytes for survival analysis. In this work, the infiltration of lymphocytes in the tumour region is quantified by employing normalized versions of statistical co-localization measures of Morisita-Horn^37^ and Shannon Diversity^38^ indices. Recently, some studies have used ecological measures like Morisita-Horn and Getis-Ord^46^ for quantifying immune-cancer co-localization and hotspot analysis. For instance, Maley *et al*.^47^ have reported the immune-cancer co-localization as a prognostic factor for breast cancer. Nawaz *et al*.^48^ have used hotspot analysis to identify the statistically significant hotspots of cancer and immune cells. Both of the above studies used handcrafted feature-based machine learning methods for identification of tumour and immune cell areas. In contrast, we have used a deep learning based approach for identifying tumour and lymphocyte rich regions at patch level which have been shown to be more accurate. Moreover, the normalized TIL abundance score is computed as a combination of lymphocyte-to-tumour ratio and their statistical co-localization in a WSI. In summary, the proposed TILAb score is a digital biomarker which is based on more accurate classification of tumour and lymphocytic regions, is motivated by the biological definition of TILs as tumour infiltrating lymphocytes, and is objective and reproducible.

**Figure 10:**
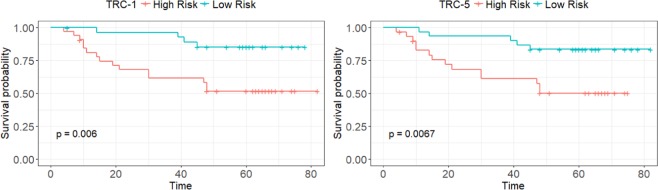
Kaplan Meier (KP) curves for disease free survival of OSCC on 3-fold cross-validation using TRC-1 and TRC-5.

The prognostic significance of this score for DFS is investigated by employing univariate and multivariate analyses using clinico-pathological parameters. We analyse the prognostic significance of the TILAb score using Cox proportional hazard model. The TILAb score shows good statistical significance in both univariate (Table 4) and multivariate (Table 6) analyses (*p* < 0.05). Therefore, the TILAb score can be used as an independent prognostic parameter in OSCC patients. The Kaplan-Meier curves showed the ability of TILAb score to stratify patents into long-term (low risk) and short-term (high risk) DFS (*p* = 0.0006). Although the main focus of the paper is on DFS, besides DFS the prognostic significance of TILAb score for overall survival is also investigated. The Kaplan Meier curves are shown in Fig. S9 in the supplementary material which illustrates that the TILAb score gives good separation for overall survival too. To highlight the independence of the proposed model on initial image selection, the 3-fold cross validation with random initial image selection is used for DFS. The KM curves and associated p-values, shown in Fig. 10, illustrate that the proposed models (TRC-1 and TRC-5) are prognostically significant for different sets of training and test splits. Moreover, to show the robustness of our method, the C-indices (with 95% confidence intervals) of prognostic models for disease free survival and overall survival are also computed and shown in Tables 5 and S2 respectively. The results of the proposed method are also in agreement with previous findings based on manual and immunohistochemistry based TIL quantification^26,49,50^ in OSCC. Fang *et al*.^50^ analysed the prognostic significance of tumour infiltrating immune cell in OSCC. The immune cells were identified by their specific markers (CD8, CD4, T-bet, CD68 and CD57). High CD8 (T-cells) and CD57 (NK-cell) expression were significantly associated with longer survival.

Hematoxylin & Eosin staining is routinely used in pathology labs around the globe in clinical practice for cancer diagnostics. Automated methods for extracting information related to TILs from the whole slide images can help in treatment planning according to the immune response. The proposed framework for automated quantification of TILs, computation of their abundance score, and its prognostic analysis of patient survival using OSCC histology images is the first of its kind. Even though the total number of cases involved in this study is limited (*n* = 70), some other studies have reported results on smaller cohorts^51^ (*n* = 48) or using tissue microarrays^52^ (TMAs), which contain much smaller snapshots of tumour /lymphocytes characteristics as compared to the whole slide images. Having said that, the results of this study need to be cross-validated on data from large multi-centric patient cohorts before they can be adopted in clinical practice.

In addition to the application to cancer resections and information about future behaviour, our proposed TILAb score can be applied to the initial biopsy specimen undertaken before surgical resection or chemoradiotherapy. A biopsy and histological assessment is the gold standard for pre-operative diagnosis and a prerequisite for staging. As part of this assessment, pathologists report the presence/absence and comment on the density of the host lymphocytic response. The TILAb score can provide an objective quantification on this initial biopsy providing vital information about prognosis to the clinical team with the potential to guide treatment decisions and risk stratification.

## Supplementary information

Supplementary Document

## Data Availability

The code of the proposed method is publicly available on github (https://github.com/TIA-Lab/TILAb-Score). We plan to release all the annotated data in the public domain, should this paper be accepted for publication.
