# Supplementary material for: A Novel Digital Score for Abundance of Tumour Infiltrating Lymphocytes Predicts Disease Free Survival in Oral Squamous Cell Carcinoma
Source: Sci Rep. 2019 Sep 16;9:13341. doi: 10.1038/s41598-019-49710-z (PMC6746698; doi:10.1038/s41598-019-49710-z)
Supplement: Supplementary file 1 — Supplementary Document [file 41598_2019_49710_MOESM1_ESM.pdf]

A Novel Digital Score for Abundance of Tumour Infiltrating Lymphocytes Predicts Disease Free Survival in Oral Squamous Cell Carcinoma

Muhammad Shaban[1], Syed Ali Khurram[2], Muhammad Moazam Fraz[1,3,4], Najah Alsubaie[1,5], Iqra Masood[6], Sajid Mushtaq[6], Mariam Hassan[6], Asif Loya[6], and Nasir M Rajpoot[1,4,7,*]

[1]Department of Computer Science, University of Warwick, Coventry, CV47AL, UK
[2]School of Clinical Dentistry, University of Sheffield, UK
[3]School of Electrical Engineering and Computer Science, National University of Sciences and Technology, H-12, Islamabad, Pakistan
[4]The Alan Turing Institute, NW1 2DB, London, UK
[5]Department of Computer Science, Princess Nourah University, KSA
[6]Shaukat Khanum Memorial Cancer Hospital Research Centre, Lahore, Pakistan
[7]University Hospitals Coventry, Department of Pathology, Warwickshire, UK
[*]corresponding author: n.m.rajpoot@warwick.ac.uk

1 TILAbundance (TILAb) Score

TILAb score is proposed to quantify the abundance of lymphocytes w.r.t tumour. It is defined as the product of lymphocyte-to-tumour ratio and their co-localization. The formulation of TILAb score using Morisita-Horn index as colocalization measure is given below,

$$T = \begin{cases} \frac{\sum_{i=1}^{N}(p_i^l \times p_i^t)}{\sum_{i=1}^{N}(p_i^l)^2 + \sum_{i=1}^{N}(p_i^t)^2} \times \frac{\sum_{i=1}^{N}(p_i^l)}{\sum_{i=1}^{N}(p_i^t)}, & \text{if } \sum_{i=1}^{N}(p_i^t) > 0 \\ 1, & \text{otherwise} \end{cases} \tag{1}$$

where p_i^l and p_i^t represents the percentage of lymphocyte and tumour in i^{th} cell of a grid whereas N denotes the total number of cells in a grid. The percentage of lymphoctes in each cell is independent of other cells. The Figure 1 shows the distribution of TILAb score for two different grids. The TILAb score increases with the increase in percentage of lymphocytes in grid-cells. A function can only be monotonically increasing if it has positive slope (derivative). We drive the slope of function T for $N = 1$ by taking its first derivative to show the monotonic property. For the sake of notional simplicity we replace the p_i^l and p_i^t in Eq. 1 with l and t, respectively.

$$\frac{dT}{dl} = \frac{d}{dl}\frac{(l \times t)}{(l^2 + t^2)} \times \frac{l}{t} \tag{2}$$

As t represents the probability of tumour in a grid-cell which is equivalent to 1 minus probability of lymphocyte $(1-l)$ in the same grid-cell. So t is dependent of l therefore we replace the t in Eq. 2 with $1-l$ to get following univariate equation.

$$\frac{dT}{dl} = \frac{d}{dl}\frac{(l) \times \cancel{(1-l)}}{(l^2 + (1-l)^2)} \times \frac{l}{\cancel{(1-l)}}$$

$$\frac{dT}{dl} = \frac{d}{dl}\frac{l^2}{(1 - 2l + 2l^2)}$$

$$\frac{dT}{dl} = \frac{(2l^2 - 2l + 1)\frac{d}{dl}(l^2) - (l^2)\frac{d}{dl}(1 - 2l + 2l^2)}{(1 - 2l + 2l^2)^2}$$

$$\frac{dT}{dl} = \frac{(2l^2 - 2l + 1)(2l) - (l^2)(0 - 2 + 4l)}{(1 - 2l + 2l^2)^2}$$

$$\frac{dT}{dl} = \frac{\cancel{4l^3} - 4l^2 + 2l + 2l^2 - \cancel{4l^3}}{(1-2l+2l^2)^2}$$

$$\frac{dT}{dl} = \frac{2l - 2l^2}{(1-2l+2l^2)^2}$$

$$\frac{dT}{dl} = \frac{2l(1-l)}{(1-2l(1-l))^2} \tag{3}$$

Substituting $(1-l)$ back to t in Eq. 3

$$\frac{dT}{dl} = \frac{2 \times l \times t}{(1-2 \times l \times t)^2} \tag{4}$$

As t and l are non-negative real numbers and the denominator of Eq. 4 has a square on it therefore Eq. 4 always results in positive real number. Hence the slope (derivative) of function in Eq. 2 remains positive for all value of lymphocyte percentages.

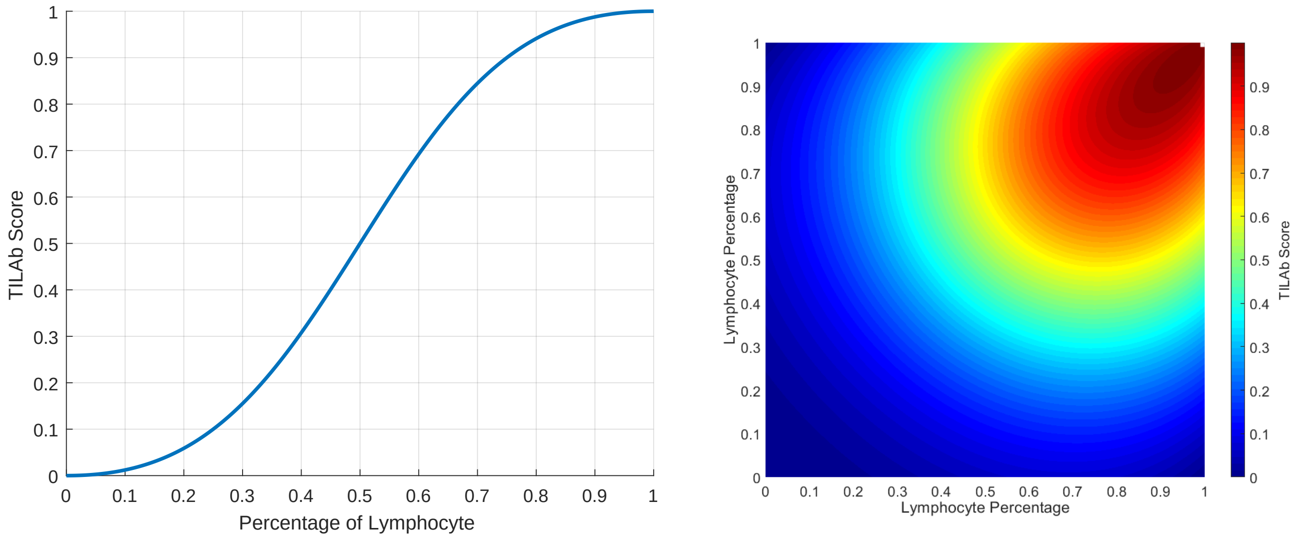

a b

Figure S 1. Both figures show the distribution of TILAb score w.r.t lymphocyte percentage in a grid.(**a**) TILAb score curve based on a simplest grid with only one cell. (**b**) TILAb score map for a grid with 2 cells. Lymphocyte percentage in each cell is independent of other cell.

2 Supplementary Tables & Figures

Table S 1. Tissue region classification results of five different classifiers.

Method	Class	Accuracy	Sensitivity	Specificity	F1-score	AUC
TRC-1	Stroma	94.33%	88.92%	96.12%	88.62%	98.07%
	Non-ROI	91.64%	85.19%	93.64%	82.82%	96.58%
	Tumour	92.11%	81.76%	95.92%	84.81%	97.37%
	Lymphocyte	98.06%	96.90%	98.44%	96.09%	99.49%
TRC-2	Stroma	95.12%	91.70%	96.21%	90.08%	98.33%
	Non-ROI	94.96%	90.74%	96.33%	89.82%	98.43%
	Tumour	94.26%	87.78%	96.47%	88.62%	98.14%
	Lymphocyte	97.25%	92.97%	98.74%	94.59%	99.34%
TRC-3	Stroma	96.00%	92.99%	96.97%	91.90%	98.95%
	Non-ROI	94.59%	85.18%	98.22%	89.76%	98.75%
	Tumour	94.17%	91.15%	95.08%	87.92%	97.89%
	Lymphocyte	98.62%	98.30%	98.72%	97.21%	99.83%
TRC-4	Stroma	95.30%	96.40%	95.00%	89.98%	98.83%
	Non-ROI	95.67%	89.91%	97.69%	91.50%	98.58%
	Tumour	94.42%	85.88%	97.59%	89.28%	98.27%
	Lymphocyte	98.37%	96.45%	99.02%	96.76%	99.63%
TRC-5	Stroma	96.05%	93.25%	96.95%	92.00%	99.02%
	Non-ROI	95.99%	93.45%	96.80%	91.85%	98.75%
	Tumour	95.12%	88.69%	97.37%	90.42%	98.50%
	Lymphocyte	98.10%	95.27%	99.06%	96.23%	99.35%

Table S 2. C-Indices (with 95% CI) of TRC-1 and TRC-5 based prognostic models for OS at different grid-cell sizes (smallest to largest).

Grid-Cell	TRC-1						TRC-5					
	TILAb-MH			TILAb-SD			TILAb-MH			TILAb-SD		
	C-Index	Lower CI	Upper CI	C-Index	Lower CI	Upper CI	C-Index	Lower CI	Upper CI	C-Index	Lower CI	Upper CI
1	0.7826	0.6158	0.9494	0.7826	0.6158	0.9494	0.7764	0.5884	0.9644	0.7826	0.5957	0.9695
2	0.7888	0.6280	0.9497	0.7826	0.6212	0.9440	0.7826	0.6055	0.9598	0.7826	0.6005	0.9647
3	0.7702	0.6020	0.9384	0.7640	0.5914	0.9365	0.7826	0.6055	0.9598	0.7888	0.6122	0.9655
4	0.7516	0.5686	0.9345	0.7578	0.5854	0.9302	0.7764	0.5981	0.9547	0.7888	0.6122	0.9655
5	0.7516	0.5752	0.9279	0.7516	0.5710	0.9321	0.7702	0.5868	0.9536	0.7764	0.5940	0.9588
6	0.7453	0.5596	0.9311	0.7516	0.5670	0.9361	0.7764	0.5973	0.9555	0.7764	0.5924	0.9604
7	0.7516	0.5662	0.9369	0.7516	0.5662	0.9369	0.7702	0.5917	0.9486	0.7640	0.5742	0.9537
8	0.7453	0.5588	0.9319	0.7453	0.5533	0.9374	0.7640	0.5830	0.9449	0.7888	0.6208	0.9569

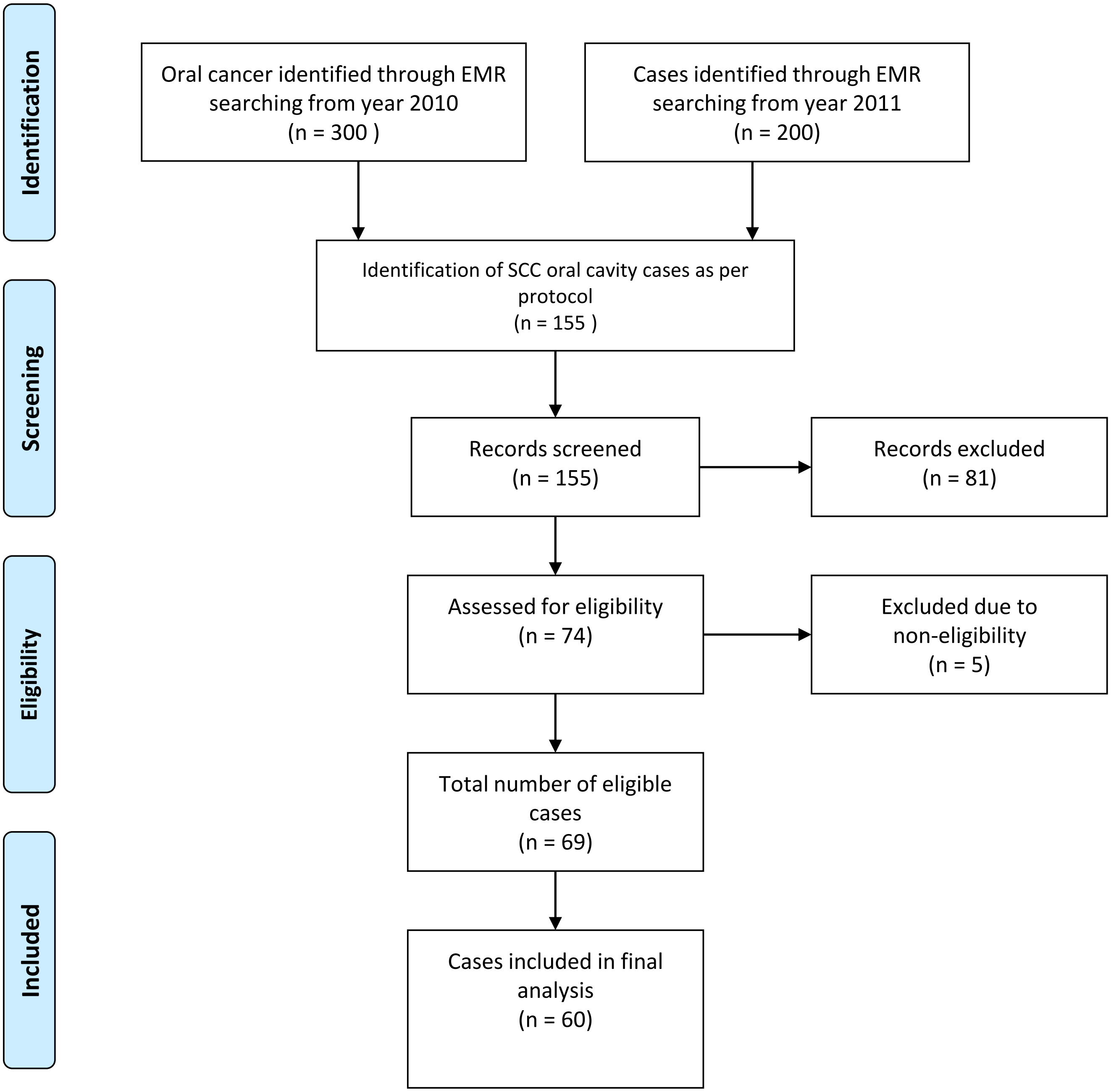

Figure S 2. PRISM flow diagram for patient selection. Eligible cases are those cases that underwent complete tumour resection with or without lymph node dissection and for which survival data were available. The cases excluded were those where either a complete resection was not done as this was needed to report all parameters and those where survival follow up of less than 3 years

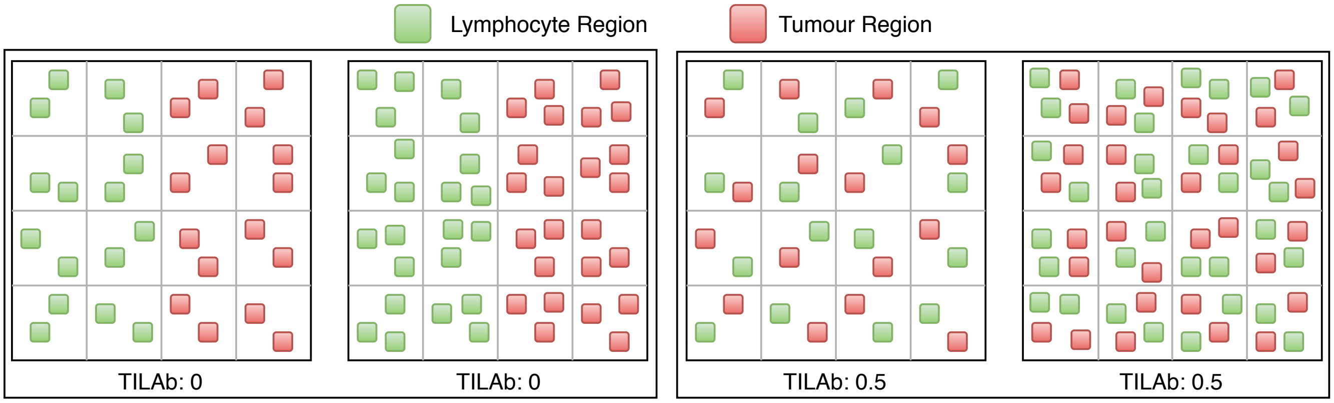

Figure S 3. The illustration of TILAb score's invariance to tumour and lymphocyte density patterns. Each pair of images has varying tumour and lymphocyte density but has same TILAb score.

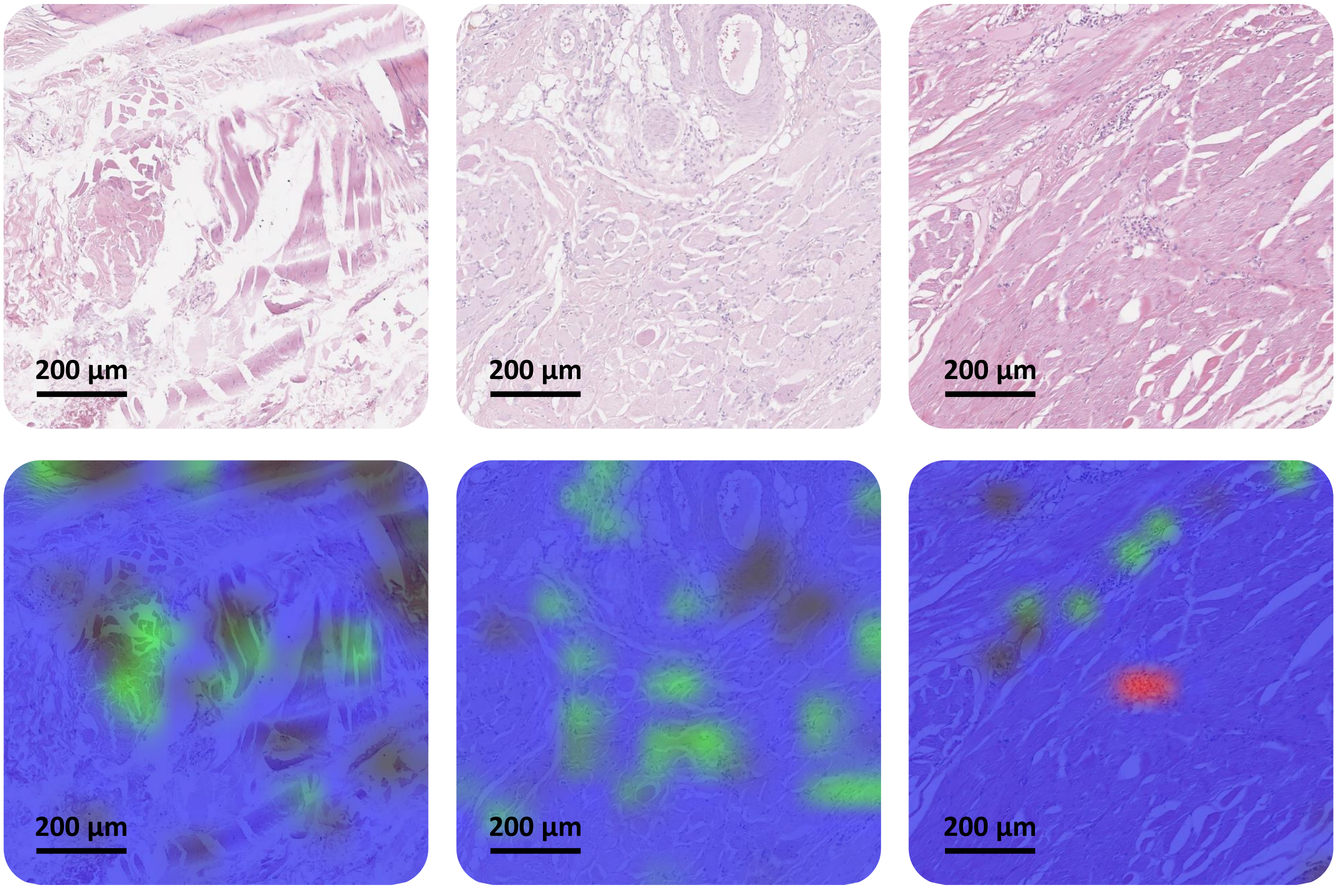

Figure S 4. Tissue region classification results (failure cases) by TRC-5 where tumour, lymphocytic, stromal and non-ROI regions are represented by red, green, blue and black colours, respectively.

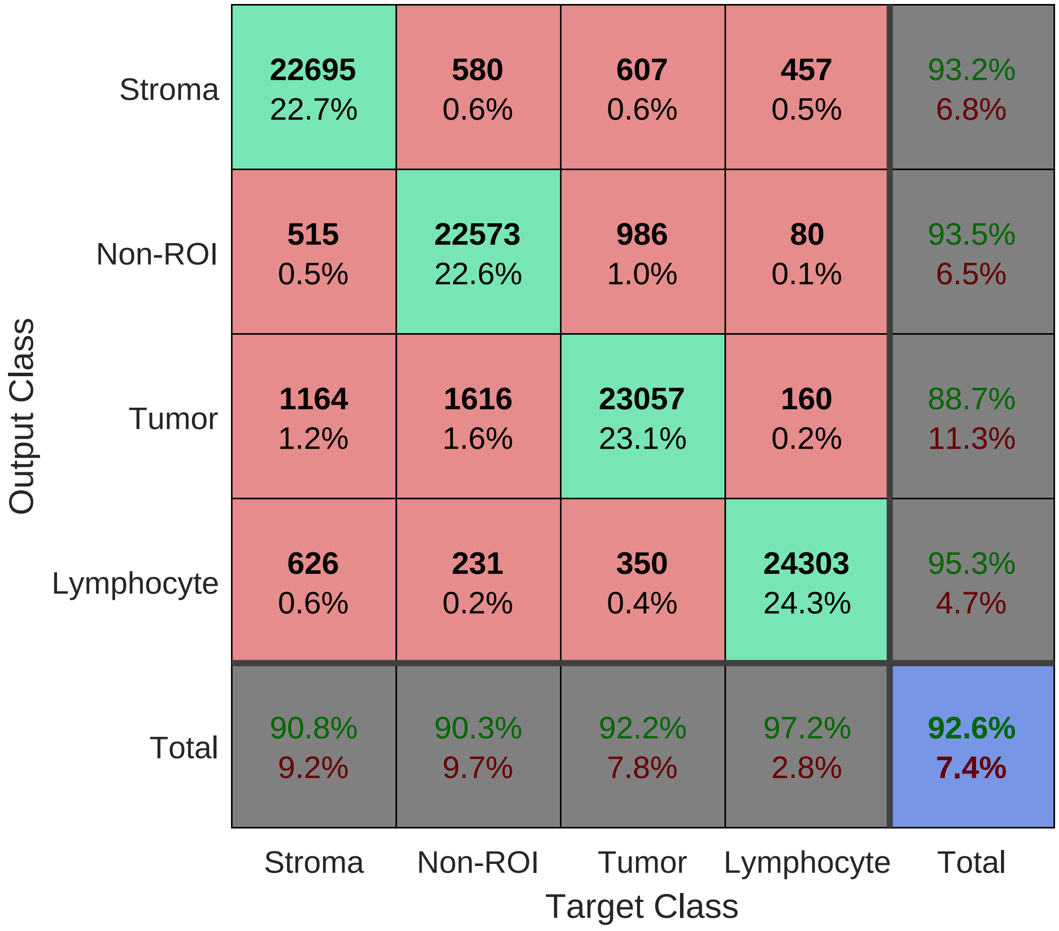

Figure S 5. Confusion matrix for all four classes using best performing tissue region classifier model.

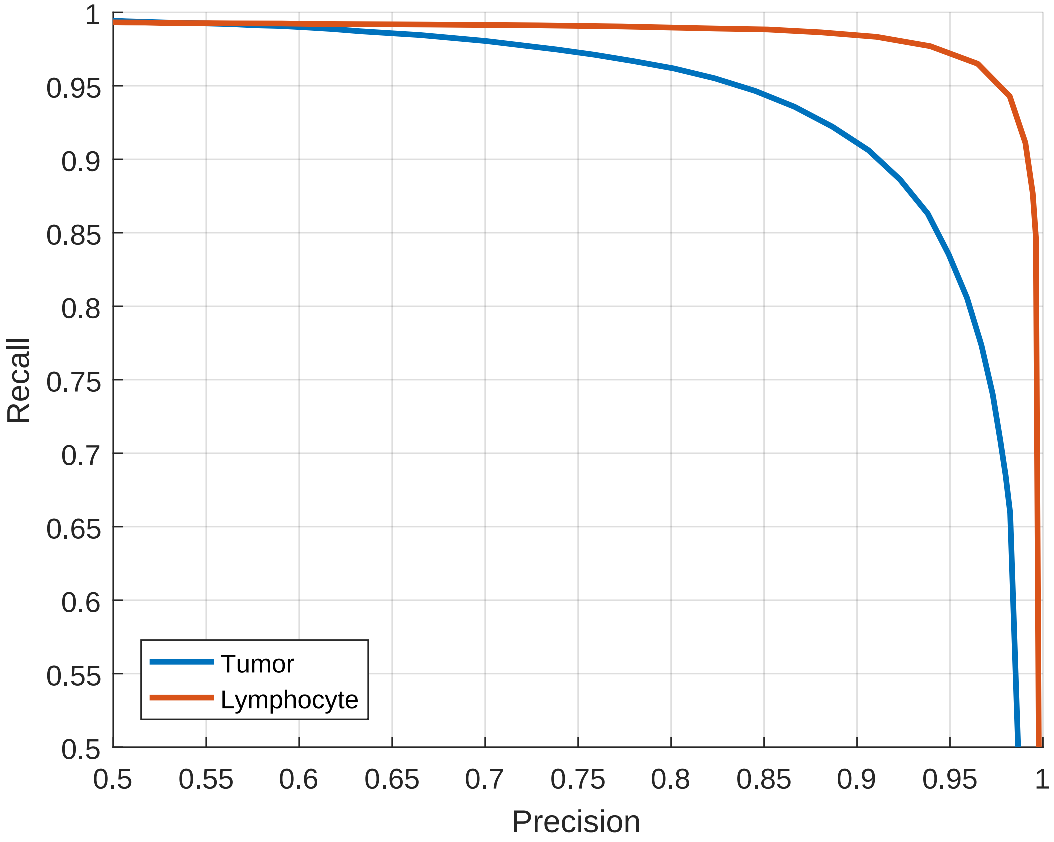

Figure S 6. Precision-Recall curves for tumour and lymphocytic region classification using the TRC-5 network.

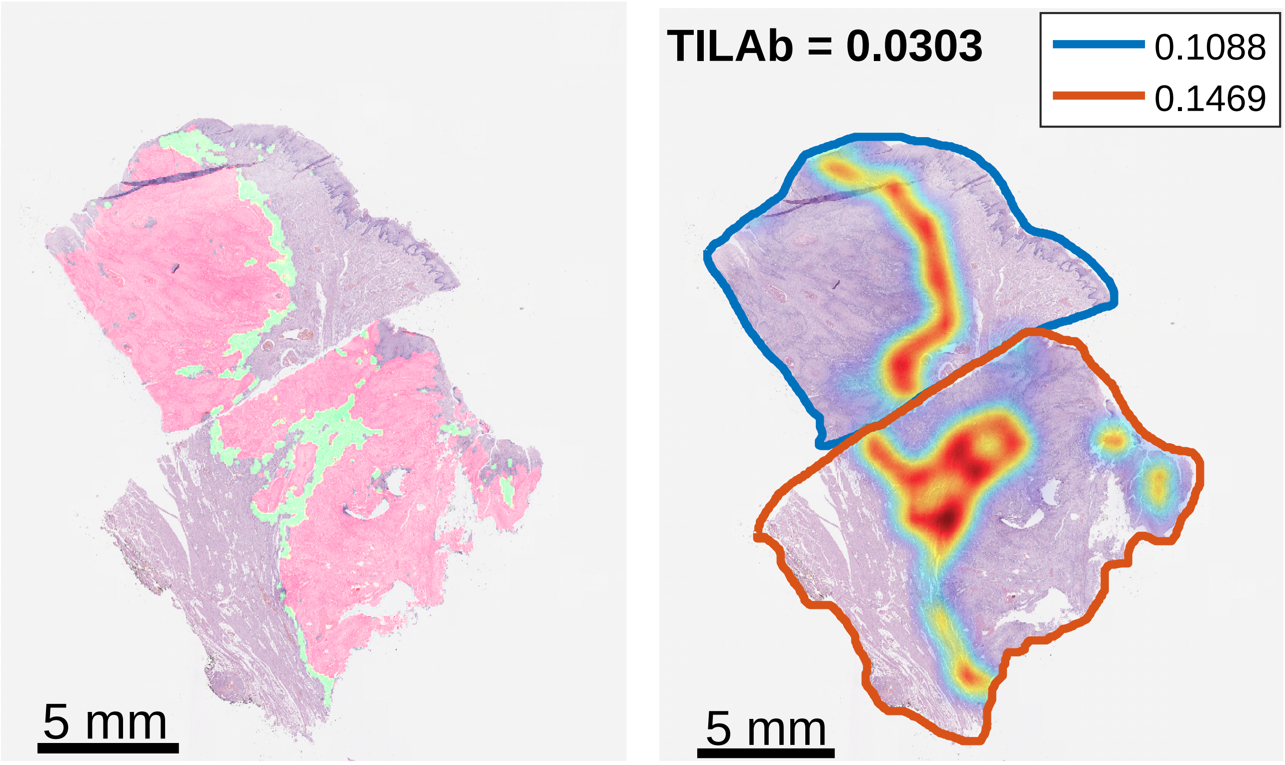

a b

Figure S 7. (**a**) Whole slide image at low resolution (1.5×) with tumour and lymphocytic region predictions overlaid in red and green colour s, respectively. (**b**) Tumour-lymphocyte co-localization map along with co-localization score for each tissue section in upper right corner and WSI level TILAb score. Colour codes maps the co-localization score to respective tissue sections.

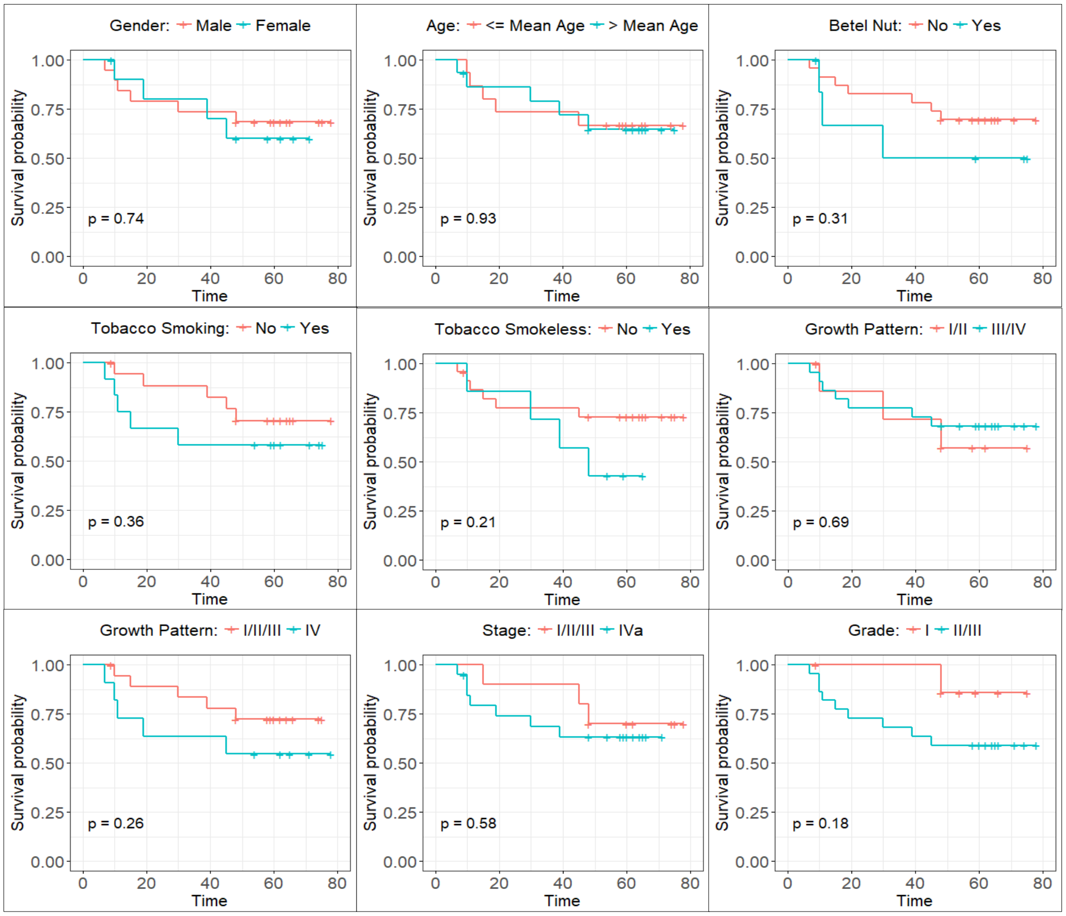

Figure S 8. Kaplan Meier (KP) curves for disease free survival of OSCC on test subset.

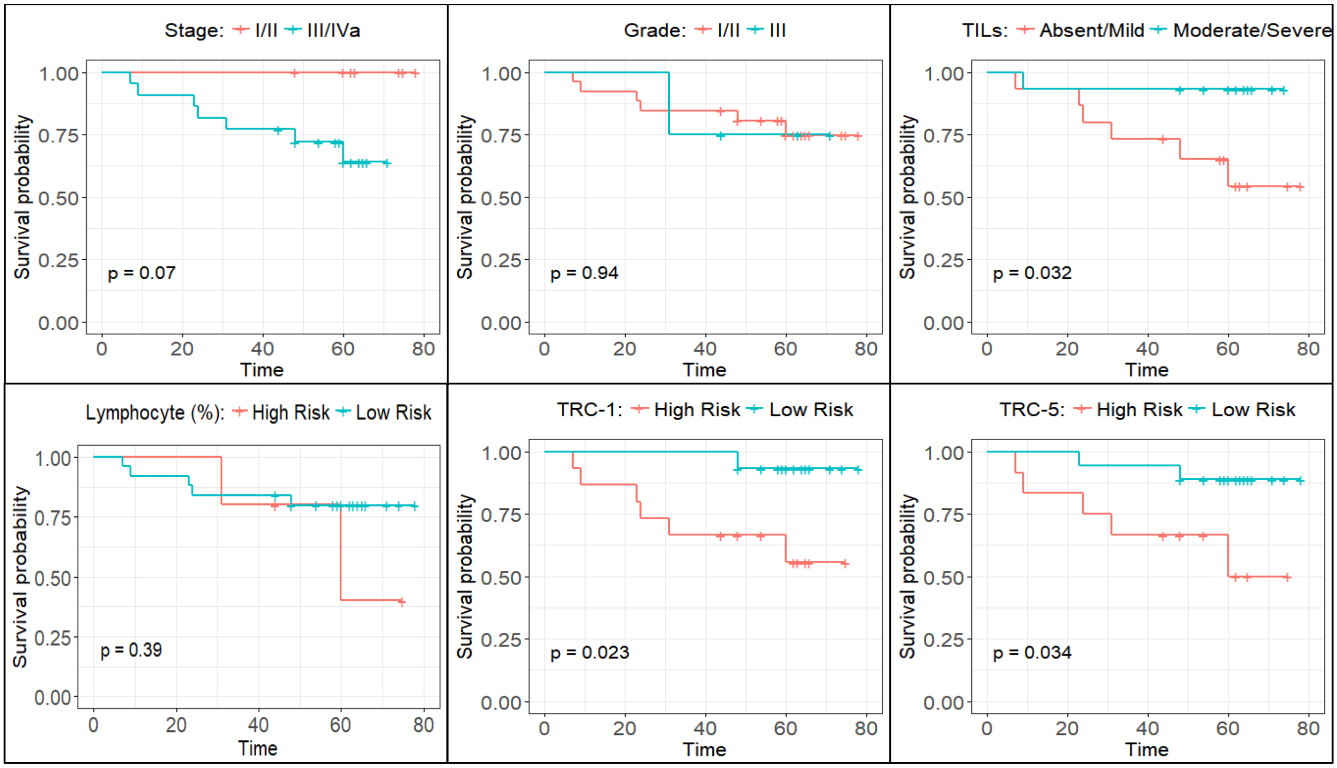

Figure S 9. Kaplan Meier (KP) curves for overall survival of OSCC on test subset. Top row contains the KP curves for pathological parameters (stage, grade and manual TIL quantification) whereas bottom bottom row shows the KP curves of digital parameters (Lymphocyte percentage in WSI, TILAb score using TRC-1 and TRC-5). The optimal cut-point values for digital parameters are 0.017, 0.124 and 0.137, respectively.
